# Decadal extreme drought reduces alpine subsoil carbon stocks

**DOI:** 10.1073/pnas.2517468123

**Published:** 2026-02-20

**Authors:** Ronglei Zhou, Jinsong Wang, Quancheng Wang, Ning Liu, Chenglong Ye, Jingjing Shi, Mengjie Liu, Zhangwei Gao, Houkun Chu, Zhenrui Zhang, Bin Niu, Song Wang, Ruiyang Zhang, Dashuan Tian, Shuli Niu

**Affiliations:** ^a^Key Laboratory of Ecosystem Network Observation and Modeling, Institute of Geographic Sciences and Natural Resources Research, Chinese Academy of Sciences, Beijing 100101, China; ^b^Department of Environment and Resources, University of Chinese Academy of Sciences, Beijing 100049, China; ^c^Sichuan Zoige Alpine Wetland Ecosystem National Observation and Research Station, Institute of Geographic Sciences and Natural Resources Research, Chinese Academy of Sciences, Beijing 100101, China; ^d^College of Resources and Environmental Sciences, Nanjing Agricultural University, Nanjing 210095, China; ^e^College of Forestry, Beijing Forestry University, Beijing 100083, China; ^f^College of Juncao Science and Ecology, Fujian Agriculture and Forestry University, Fuzhou 350000, China

**Keywords:** extreme drought, SOC stocks, mineral-associated organic C, subsoil

## Abstract

Grassland soil organic carbon (SOC) stocks are increasingly vulnerable to intensified drought. Yet, how long-term, especially extreme, drought affects these C reserves across soil profiles remains unresolved. Here, we present the response of SOC stocks at various depths (0 to 60 cm) using an experiment involving a 10-year drought gradient (P, 1/2 P, 1/4 P, and 1/12 P, where P is ambient precipitation) in an alpine grassland. We found that extreme drought (1/12 P) caused significant SOC losses in the subsoil, whereas mild to moderate droughts had no significant effects on SOC at any depth. Under extreme drought, SOC stocks declined by 27% in the 20 to 30 cm layer and by 37% in the 30 to 40 cm layer, while no significant changes were observed in the topsoil (0 to 20 cm) and the deeper layer (40 to 60 cm). Subsoil C losses were primarily driven by reductions in mineral-associated organic C (MAOC) rather than particulate organic C. Multiyear extreme drought disrupted soil–microbe–mineral interactions, as indicated by reductions in soil nitrogen availability, microbial biomass, carbon use efficiency, and mineral-binding agents. These changes collectively undermined the formation and stabilization of MAOC. Our findings suggest that prolonged extreme drought can weaken subsoil C storage and stability, highlighting the need to incorporate subsoil processes into Earth System Models to better predict soil C–climate feedback under future drought scenarios.

## Significance

Grasslands store more than 30% of global soil organic C, which is highly susceptible to drought. However, no study has quantitatively assessed how soil-profile C stocks respond to long-term, especially extreme drought. In a 10-year field experiment, we show that decade-long extreme drought decreases subsoil C stocks by 27 to 37% relative to controls, driven by reductions in mineral-associated organic C. These persistent C losses in subsoil are mainly governed by abiotic constraints on microbial functioning and the limited supply of microbial precursors. Our findings challenge the prevailing assumption that subsoil C reservoirs are resistant to drought and underscore the imperative to incorporate refined subsoil processes into Earth System Models to accurately capture soil C–climate feedback under intensifying drought.

Grasslands cover 40.5% of the Earth’s land surface (1, 2) and contain over 30% of global carbon (C) stocks, with approximately 90% of this C stored belowground as root biomass and soil organic matter (2, 3). This substantial C reservoir makes grasslands a pivotal component in the global C cycle, regulating atmospheric CO_2_ levels (4, 5). However, grassland ecosystems are highly sensitive to climate change, particularly to the increasing frequency and intensity of drought (6–8). Ongoing drought is expected to alter plant diversity, above- and below-ground C inputs, soil properties, and microbial processes, leading to complex effects on SOC in C-sequestering grasslands (9–11). Thus, understanding how drought influences SOC in grasslands has received increasing attention, as even small changes in SOC stocks can have profound consequences for soil fertility and global C cycling.

Despite growing interest, empirical results on SOC responses to drought remain highly uncertain. For instance, a 50% reduction in precipitation showed no significant effect on SOC across various grassland types (12), whereas a similar reduction in precipitation at the continental scale led to a notable increase in SOC during the second year of drought (13). These discrepancies underscore the complexity of SOC responses to drought and critical gaps in current research. One major limitation is that many studies have treated SOC as a homogeneous entity, overlooking the fact that different SOC fractions may respond in diverse ways to drought. SOC is not a uniform pool, but rather consists of distinct fractions with different formation and stabilization pathways. Particulate organic C (POC), which is largely derived from plant residues, decomposes rapidly and is highly responsive to variations in plant productivity. In contrast, mineral-associated organic C (MAOC) forms through microbial processing and organo–mineral interactions, conferring longer residence times and contributing to persistent SOC storage (14–16). While some studies suggest that POC is more vulnerable to drought due to its reliance on plant inputs (13, 17), others report that MAOC can be equally sensitive when drought disrupts microbial precursors and mineral binding (18). These findings imply that POC and MAOC may respond divergently to drought, yet there is little field-based evidence to support this. A mechanistic understanding of how SOC fractions respond to drought is essential for improving predictions of SOC stability under future climate change.

The uncertainty in SOC responses to drought is also compounded by insufficient knowledge surrounding subsoil C dynamics. While most studies focus on surface SOC, deeper soil layers remain poorly understood, despite nearly half of global SOC being stored below 20 cm (19, 20). Surface SOC turns over rapidly and is primarily influenced by plant productivity and aboveground litter. In contrast, subsoil organic carbon is assumed to be stable, regulated mainly by root-derived C, soil aeration, and microbial activity (19, 21). Although climate impacts on SOC generally weaken with depth, microbial activity, and mineral interactions become increasingly important for stabilizing SOC in deeper layers (22, 23). Drought can alter soil moisture, microbial attributes, mineral protection, and root distribution differently at different soil depths, potentially leading to divergent effects on surface and deeper SOC fractions (24, 25). However, it remains unclear how these depth-specific processes affect the balance between fast-cycling, POC-dominated surface soils and slow-turnover, MAOC-rich subsoils (26), thereby influencing the overall SOC stability. The lack of depth-resolved field data on the responses of SOC fractions to drought hinders accurate assessments of SOC vulnerability under projected drought scenarios.

The issue of SOC responses to drought is further exacerbated by the combined effects of drought intensity and duration (8, 27, 28). Previous studies have typically imposed short-term droughts with uniform intensity, typically involving a 50% reduction in precipitation, or rely on seasonal natural drought events (12, 29, 30). These studies often overlook the differential effects of varying drought intensities and the cumulative effects of prolonged droughts (31, 32). Yet, the sensitivity of SOC likely varies with drought severity and duration. Moderate drought can stimulate plant compensatory growth through physiological and morphological adaptations, potentially sustaining or even enhancing aboveground C inputs to the soil (33, 34). However, extreme drought can significantly reduce plant productivity or even cause mortality (8, 35). The different survival strategies of plants in response to various drought severities may further alter belowground C allocation and consequently SOC responses. Moreover, recent projections indicate that extreme droughts are becoming more frequent and widespread, with multiyear cumulative droughts exerting the most profound impacts on grasslands (8). However, few long-term experiments have investigated how drought intensity shapes the responses of SOC and its fractions at different soil depths, leaving major uncertainties in predicting SOC dynamics under future drought extremes.

To address these knowledge gaps, we conducted a decade-long rainfall manipulation experiment (2014–present) in alpine grasslands on the Tibetan Plateau (36, 37). This experiment simulated various drought intensities (5/4 P, P, 3/4 P, 1/2 P, 1/4 P, and 1/12 P, where P is the ambient precipitation), offering a unique opportunity to investigate responses of SOC stocks across the entire soil profile (0 to 60 cm) to the drought gradient. Specifically, we partitioned SOC into POC and MAOC and evaluated how their stocks varied with soil depth and drought severity. We further examined the potential mechanisms regulating SOC stability under drought, including soil nutrient and moisture availability, microbial attributes, and mineral protection. Our overarching goal was to determine whether long-term drought alters SOC stocks across soil profiles and to identify mechanisms that could destabilize SOC pools.

## Results

### Change in SOC Stocks across Soil Profiles under Drought Conditions.

**Fig. 1.**
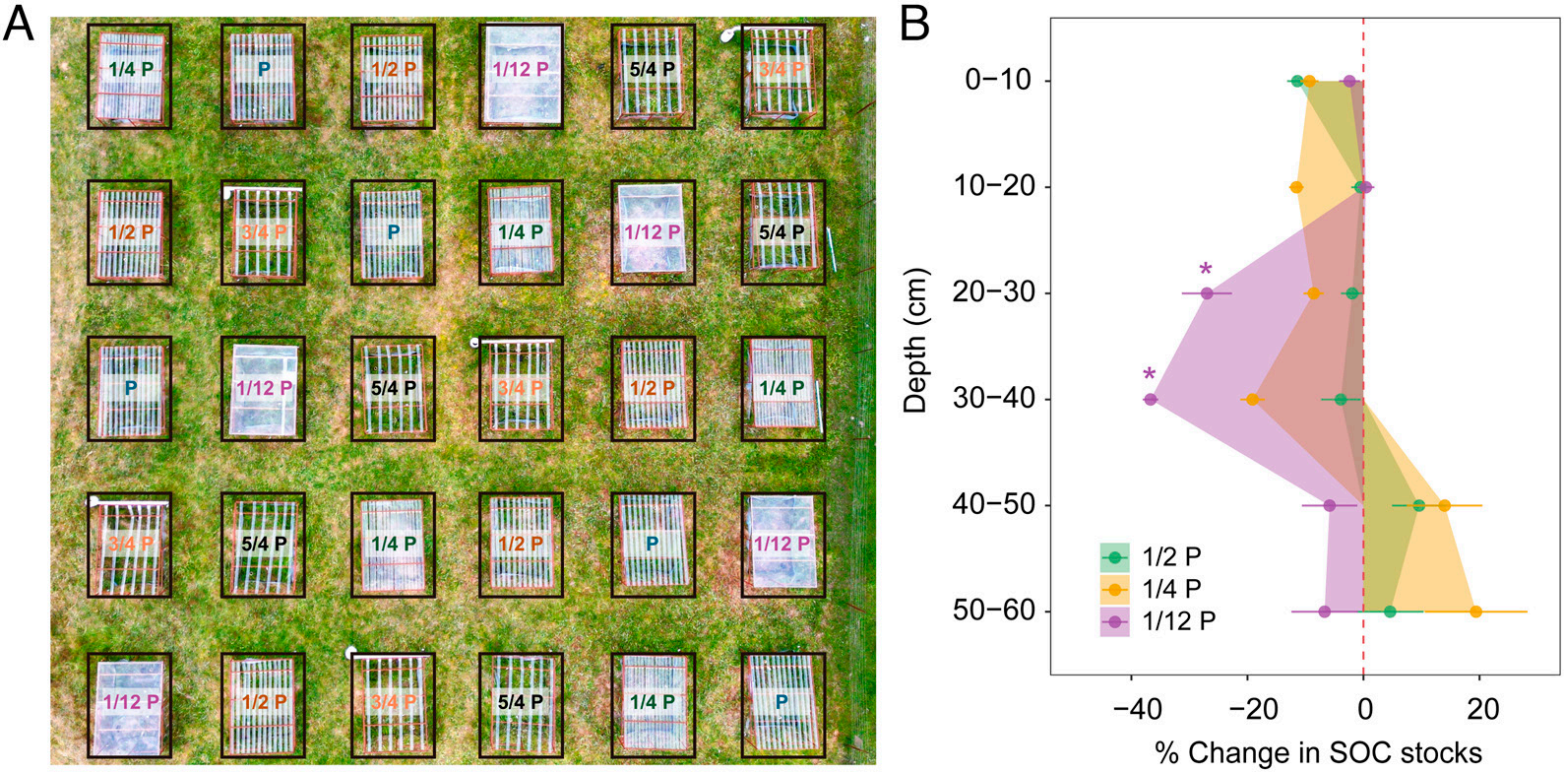
Response of soil organic carbon (SOC) stocks to drought treatments. (*A*) The field drought experimental plots at the study site. (*B*) Responses of SOC stocks across soil profiles under various drought intensities, where P is ambient precipitation. The mean and SEM of the difference in SOC stocks between drought and control plots, expressed in % [(Drought treatment − Control)/Control × 100].

Significant SOC losses were observed under extreme drought (1/12 P) in the 20 to 30 cm and 30 to 40 cm subsoil layers. In contrast, SOC stocks were not changed by mild to moderate drought intensities at any depth (Fig. 1*B*). Specifically, after 10 y of extreme drought, SOC stocks decreased by 27% in the 20 to 30 cm layer (from 3.10 ± 0.14 kg C m^−2^ to 2.32 ± 0.44 kg C m^−2^, mean ± 95% CI) and by 37% in the 30 to 40 cm layer (from 2.03 ± 0.21 kg C m^−2^ to 1.28 ± 0.15 kg C m^−2^) (*SI Appendix*, Fig. S2*C*). In contrast, SOC stocks in topsoil (0 to 10 cm and 10 to 20 cm) and deeper soil (40 to 50 cm and 50 to 60 cm) remained relatively stable. Losses in SOC stocks were primarily driven by changes in SOC concentration rather than bulk density, since bulk density remained constant across all drought treatments, while changes in SOC concentration aligned with variations in SOC stocks (*SI Appendix*, Fig. S2).

**Fig. 2.**
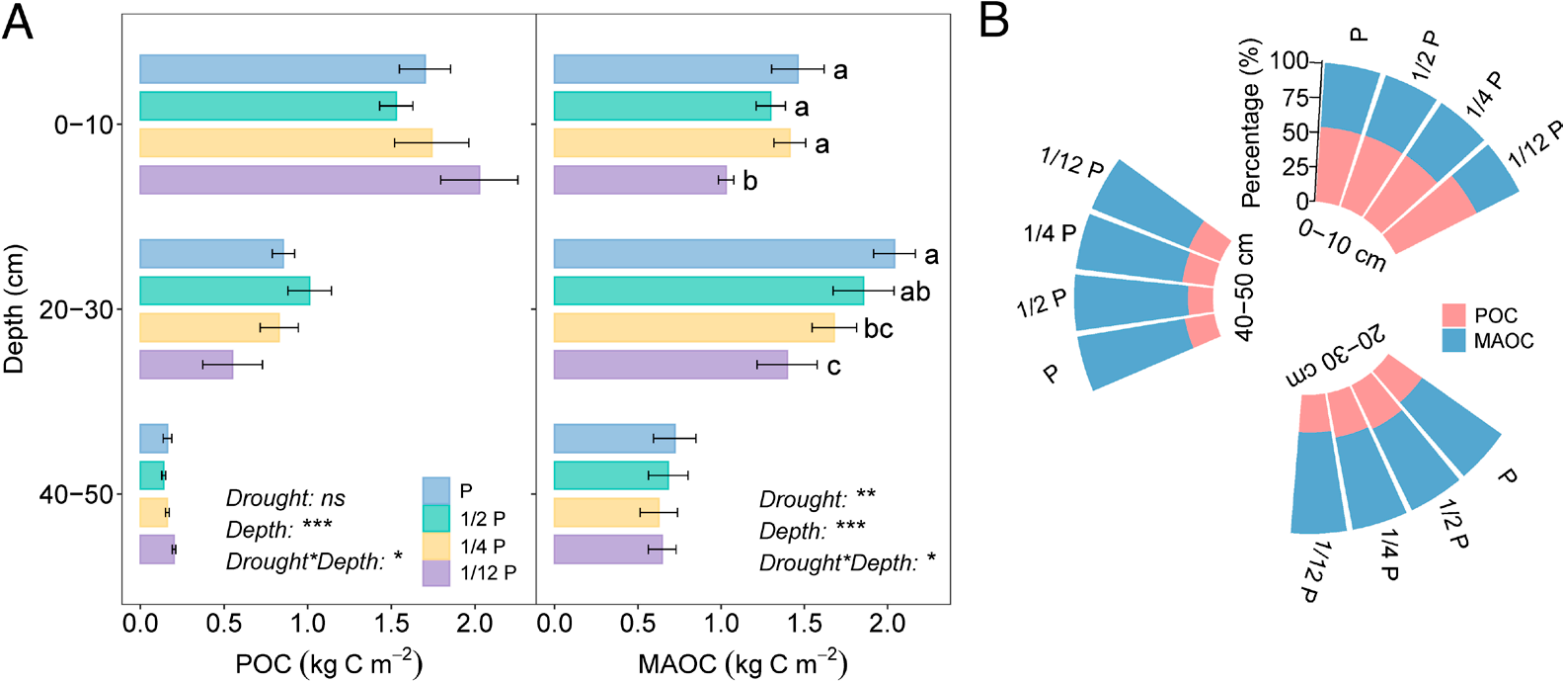
Responses of particulate organic carbon (POC) and mineral-associated carbon (MAOC) to experimental drought. (*A*) Changes in POC and MAOC stocks for the surface soil (0 to 10 cm), subsoil (20 to 30 cm), and deep soil (40 to 50 cm) under drought treatments. Data are mean values ± SEM (*n* = 5). Different letters on the top of bars represent significant differences among drought treatments. Statistical analysis was performed by linear mixed-effects models at the significance level of 0.05. (*B*) Proportional distribution of POC and MAOC within soil organic carbon pools under drought treatments. ***P < 0.001, **P < 0.01, *P < 0.05, *ns*: no significant effect.

We further investigated the effect of drought on SOC fractions by separating SOC into particulate organic carbon (POC) and mineral-associated organic carbon (MAOC) in surface soil (0 to 10 cm), subsoil (20 to 30 cm), and deep soil (40 to 50 cm) (*Materials and Methods*). Our results revealed that the subsoil C depletion under extreme drought was mainly due to reductions in MAOC. Specifically, under the extreme drought, the amount of MAOC in both the surface soil and the subsoil decreased significantly, by 0.43 ± 0.07 kg C m^−2^ and 0.65 ± 0.17 kg C m^−2^, respectively (Fig. 2*A*). In contrast, POC did not change across drought intensities, indicating a limited role in mediating drought-induced SOC changes.

The dominant role of MAOC in shaping SOC responses to drought was further supported by its increasing contribution to the total SOC pool with depth. MAOC accounted for 41% of total SOC in the surface soil, 65% in the subsoil, and 68% in the deep soil (Fig. 2*B*). Notably, under long-term drought conditions, the proportion of MAOC decreased in the surface soil but increased in the subsoil and deep soil layers, suggesting a depth-dependent reorganization of SOC fractions. The relationship between MAOC and SOC stocks also strengthened with depth, with no significant correlation in the surface soil, but strong positive correlations in the subsoil (R^2^ = 0.71, *P* < 0.01) and deep soil (R^2^ = 0.85, *P* < 0.001) layers (*SI Appendix*, Fig. S3). These findings highlight the pivotal role of MAOC in determining drought-induced SOC losses, particularly in deeper soil layers, where its stability and dominance are essential for maintaining SOC stocks.

### Mechanisms Underlying Subsoil C Losses under Extreme Drought.

**Fig. 3.**
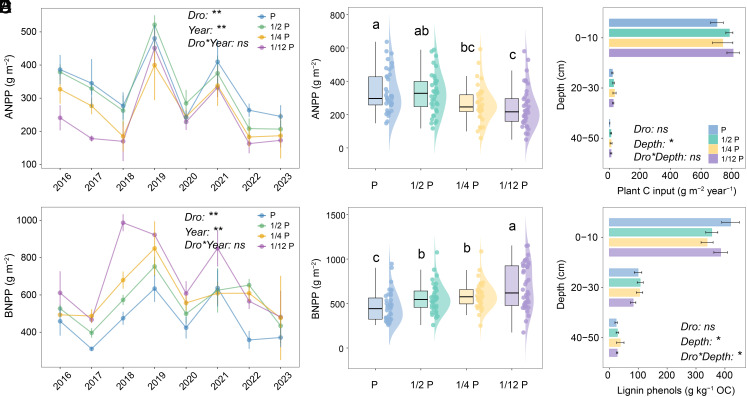
Variations in plant net primary productivity and plant carbon input across drought gradients. (*A*) Temporal changes of aboveground net primary productivity (ANPP) under different drought treatments. (*B*) Drought effects on mean ANPP over the experimental year. (*C*) Drought effects on mean plant carbon input over the course of the experiment. (*D*) Temporal changes in total belowground net primary productivity (BNPP) under various drought treatments. (*E*) Drought effects on total mean BNPP over the experimental year. (*F*) Drought effects on lignin phenols measured in 2023. Data are mean values ± SEM (*n* = 5). Box boundaries represent the 75th and 25th quantiles, and whiskers represent 1.5 times the interquartile range. Lines in the boxplot represent the median value. Different lowercase letters on the top of boxes represent significant differences among drought treatments. Statistical analysis was performed by linear mixed-effects models at the significance level of 0.05. **P < 0.01, *P < 0.05, Dro: drought treatment, *ns*: no significant effect.

To elucidate the mechanisms underlying significant subsoil C losses under extreme drought, we systematically examined plant C inputs, microbial processes, and mineral protection that can shape SOC formation and stability. The decadal-long data revealed that aboveground net primary productivity (ANPP) decreased significantly with increasing drought intensity (Fig. 3 *A* and *B*), while total belowground net primary productivity (BNPP) increased (Fig. 3 *D* and *E*). This pattern indicates a shift in plant C allocation toward belowground (*SI Appendix*, Fig. S4). Plant-derived C inputs were calculated as the sum of ANPP and BNPP_topsoil_ for the 0 to 10 cm layer, and as BNPP_subsoil_ for the 20 to 30 cm layer (Materials and Methods). Nonetheless, neither plant-derived C inputs nor lignin phenols concentrations in the subsoil changed significantly (Fig. 3 *C* and *F*). These results suggest that plant C inputs are not the main cause of subsoil C losses under long-term extreme drought. Instead, subsoil C depletion likely results from disruptions in microbial functioning or mineral-associated stabilization processes.

**Fig. 4.**
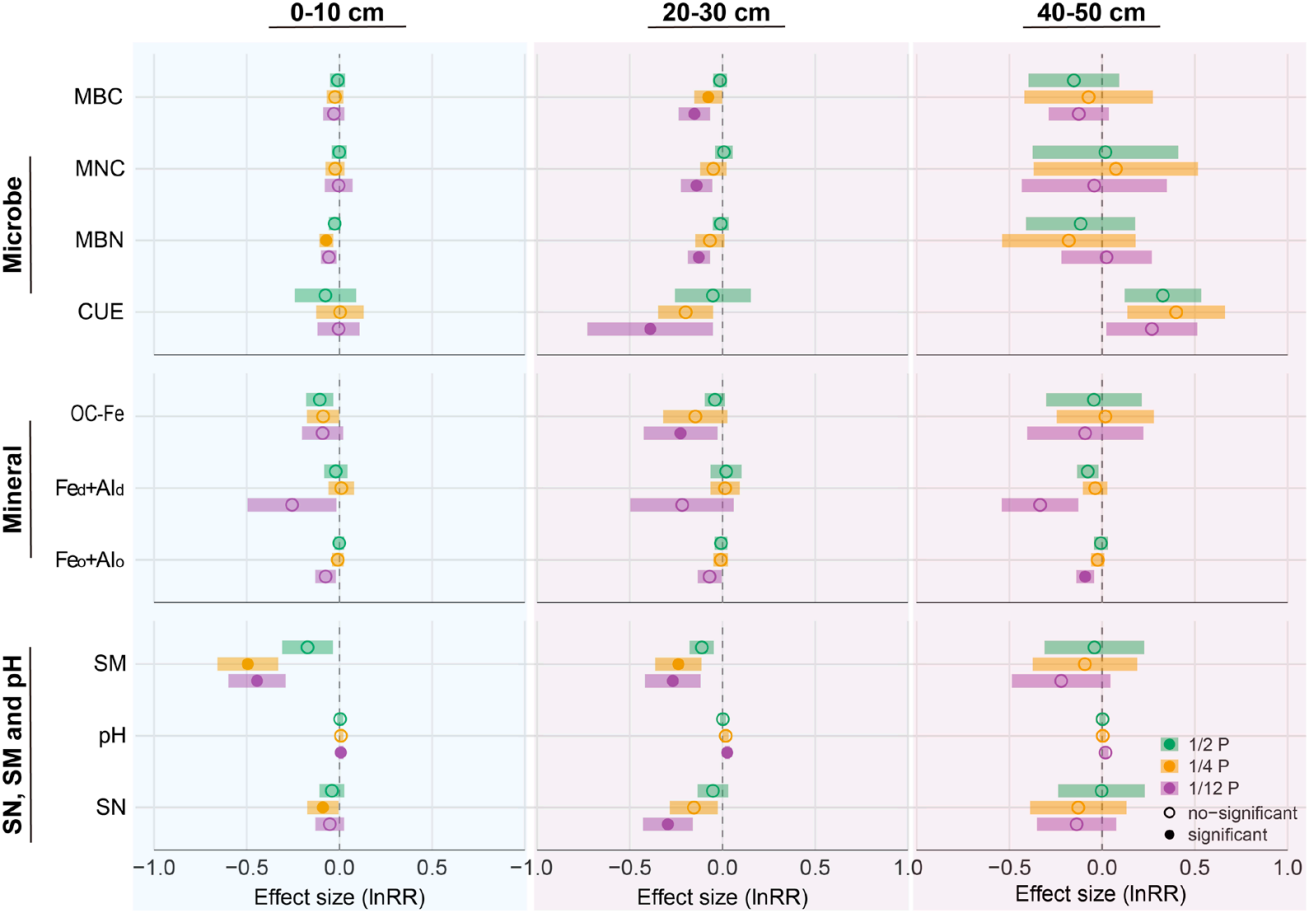
Responses of soil microbial, mineral, and physicochemical properties to drought in different soil depths. MBC: Microbial biomass carbon; MNC: Microbial necromass carbon; MBN: Microbial biomass nitrogen; CUE: Microbial carbon use efficiency; OC-Fe: Iron-bound organic carbon; Fe_o_+Al_o_: sum of oxalate-extractable Fe and Al content; Fe_d_+Al_d_: sum of dithionite-extractable Fe and Al content; SN: total soil nitrogen; SM: soil moisture. The log response ratio [LnRR = ln(Drought/Control)] and its SEM between drought and control were calculated from the mean values and their variability. Statistical analysis was performed by linear mixed-effects models at the significance level of 0.05.

Drought significantly altered microbial properties. Microbial biomass carbon (MBC), necromass carbon (MNC), and carbon use efficiency (CUE) all decreased significantly in the subsoil under extreme drought (Fig. 4 and *SI Appendix*, Fig. S6). Specifically, MBC declined by 0.16 ± 0.04 g C kg^−1^ soil, MNC by 3.89 ± 1.04 g N kg^−1^ soil, and CUE by 0.34 ± 0.24 relative to controls. These reductions in microbial biomass and residues likely limited the formation of MAOC. Spearman correlation analysis showed strong positive relationships between MAOC and these factors. Random forest model further demonstrated that MBC, MNC, and MBN contributed significantly to the variation in MAOC. Overall, microbial attributes explained 25% of the variation in MAOC (*SI Appendix*, Fig. S5). These findings highlight the critical role of microbial processes in regulating subsoil MAOC reductions under extreme drought.

The stability of MAOC was also closely linked to soil physicochemical properties and mineral protection. Specifically, SN, SM, and pH together explained 37% of the variation in MAOC, while mineral protection contributed 16% to the variation in MAOC. Under extreme drought, soil moisture (SM) and total soil nitrogen (SN) in the subsoil decreased significantly, while pH increased significantly (Fig. 4). Additionally, we observed that under extreme drought conditions, iron-bound organic C (OC-Fe) declined by 0.95 ± 0.53 g kg^−1^ (Fig. 4 and *SI Appendix*, Fig. S6). These reductions in OC-Fe and variation in soil physicochemical properties likely weakened the protective mechanisms that stabilize MAOC. This interpretation was further supported by partial correlation and random forest analysis, which identified OC-Fe, soil pH, SN, and SM as key drivers of MAOC variation (*SI Appendix*, Fig. S5).

## Discussion

Our findings indicated that mild to moderate droughts had no significant impact on SOC stocks, which is consistent with global drought manipulation experiments (50% reduction in precipitation) (12) and a global meta-analysis (38). This resilience likely results from minimal changes in soil C inputs and outputs under less severe droughts. In our study, plant C input and microbial attributes remained relatively unchanged under the mild and moderate drought treatments (Figs. 3 and 4), maintaining a near balance between C input and decomposition. Such resilience suggests that SOC can withstand moderate water stress and that significant C losses only occur when drought intensity and duration jointly exceed this resilience threshold. In support of this, we found that long-term extreme drought caused significant SOC losses in the subsoil, driven primarily by the loss of protected C. Our field experiment provides robust evidence that decadal extreme drought can disrupt the storage and stability of subsoil organic C, challenging the traditional view that deeper SOC is more resistant to climate change (22). Such subsoil C depletion represents a critical yet overlooked vulnerability in alpine grassland C pools, particularly given the projected increases in drought extremes under future climate scenarios (8, 28).

**Fig. 5.**
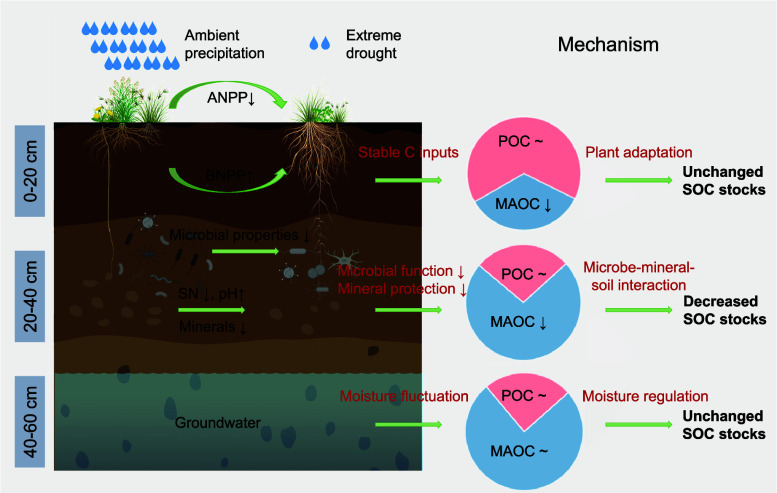
Schematic representation of depth-dependent response of SOC stocks to long-term extreme drought. Pie charts indicate the proportional distribution of POC and MAOC within SOC pools. Extreme drought represents 1/12 of ambient precipitation treatment (*Materials and Methods*). ANPP and BNPP are above- and below-ground net primary productivity, respectively. Microbial function includes microbial biomass carbon and nitrogen, microbial necromass carbon, diversity, microbial carbon use efficiency, and the first principal component (PC1) from principal component analysis (PCA) downscaling of microbial co-occurrence network parameters. Mineral protection includes dithionite-extractable Fe and Al and iron-bound organic carbon (OC-Fe). ↑ indicates an increase in the target parameter under extreme drought; ↓ indicates a decrease; and ~ represents no change.

We showed that subsoil C losses under extreme drought were primarily driven by the breakdown of soil–microbe–mineral interactions, rather than by changes in plant-derived C inputs. This was characterized by a significant decline in MAOC, while POC remained relatively stable, highlighting the dominant role of MAOC in regulating subsoil C losses. We identify at least three mechanisms underlying this pattern. First, N limitation and elevated pH could intensify subsoil MAOC losses. Drought-induced declines in soil total N and MBN (*SI Appendix*, Fig. S7) may trigger a “N mining” strategy, whereby soil microbes decompose N-rich MAOC to meet their nutrient demands (14, 17). Simultaneously, elevated pH can destabilize mineral–organic associations and suppress lignin-degrading enzymes, causing plant residues to remain in the POC pool rather than converting to MAOC (39, 40). Second, a decline in microbial functioning could have constrained MAOC formation. Reduced microbial CUE under drought may shift the microbial community toward a “starvation survival strategy” (25, 39), whereby more C is allocated to maintenance metabolism rather than biomass production. Concurrent declines in MBC, MNC, and diversity, along with simplified microbial co-occurrence networks (Fig. 4 and *SI Appendix*, Figs. S9–S11) may weaken the supply of MAOC precursors (e.g., microbial residues) and accelerate the decomposition of existing MAOC by eliminating functional redundancy (41, 42). Third, reduced mineral protection further exacerbates MAOC losses. Declines in OC-Fe indicate the low association of organic C with reactive Fe during soil drought (16). This may result from both a decrease in reactive Fe minerals that normally bind with organic ligands via cation exchange, coprecipitation, or surface sorption (43), and from increased soil pH (Fig. 4), which reduces the surface positive charge of Fe oxides and thereby weakens their electrostatic attraction to negatively charged organic molecules (44). The fact that these MAOC changes occurred despite stable plant C inputs, implying that subsoil C pool and stability depend on a delicate balance of abiotic constraints, microbial processes, and mineral protection. Our quantitative analysis (*SI Appendix*, Fig. S5) further suggests that abiotic constraints on microbial functioning and the limited supply of MAOC precursors contribute more to MAOC losses than weakened mineral protection. When drought disrupts soil–microbe–mineral interactions beyond critical thresholds, subsoil C becomes susceptible to potentially irreversible losses dominated by MAOC degradation (Fig. 5).

Unexpectedly, drought did not significantly affect SOC stocks in the 0 to 20 cm layer, which is mainly attributed to plant adaptation and C allocation strategies. Drought reduced ANPP while increasing BNPP, indicating enhanced C allocation to belowground roots (Fig. 3 and *SI Appendix*, Fig. S4). This shift agrees with previous findings that drought-stressed plants tend to allocate more photosynthates belowground for osmotic regulation and storage, thereby stabilizing plant-derived C inputs to soil (45). Moreover, multiyear drought reduced species richness (*SI Appendix*, Fig. S8), likely shifting community composition toward drought-tolerant species (46). Such species often develop root traits that increase fine-root density in shallow soils to optimize water uptake during episodic rainfall (47, 48). Root distribution data showed that 90% of fine roots were concentrated in the 0 to 20 cm layer, with drought increasing their proportion in surface soils (Table 1). This root-driven C reallocation can stabilize plant C inputs, maintaining POC stocks under drought conditions. Moreover, the absence of SOC responses in the 40 to 60 cm layer warrants further investigation. One possible explanation is that seasonal groundwater recharge buffered the effects of drought (49), as indicated by the relatively stable levels of soil moisture, microbial biomass, and plant-derived C inputs observed at these depths (Figs. 3 and 4 and *SI Appendix*, Fig. S6). Alternatively, although iron plates were installed around the plots to a depth of 40 cm, some lateral or vertical water movement may have occurred below this depth, potentially mitigating drought effects. Future research should explicitly evaluate the role of deep soil hydrology in modulating drought impacts on SOC.

**Table 1.** Root density distribution along soil profiles under drought treatments

Depth (cm)	P		1/2 P		1/4 P		1/12 P	
	RD	Per	RD	Per	RD	Per	RD	Per
0–10	2.48 ± 1.33	78.28	3.25 ± 0.91	83.36	3.24 ± 1.87	81.5	3.95 ± 1.62	84.27
10–20	0.51 ± 0.64	16.07	0.28 ± 0.09	7.15	0.36 ± 0.22	9.08	0.35 ± 0.11	7.38
20–30	0.11 ± 0.02	3.48	0.18 ± 0.06	4.63	0.19 ± 0.13	4.8	0.17 ± 0.07	3.54
30–40	0.04 ± 0.02	1.29	0.1 ± 0.08	2.49	0.11 ± 0.05	2.7	0.15 ± 0.13	3.1
40–50	0.02 ± 0.01	0.62	0.07 ± 0.07	1.76	0.05 ± 0.03	1.26	0.06 ± 0.06	1.25
50–60	0.01 ± 0.01	0.26	0.02 ± 0.03	0.61	0.03 ± 0.04	0.66	0.02 ± 0.03	0.45

P is ambient precipitation. RD: root density (g 100 cm^−3^), Per: percentage (%).

Our findings provide compelling evidence that decadal extreme droughts can cause substantial subsoil C losses, challenging the widely held assumption—based largely on short-term experiments and surface soils—that drought has a minimal impact on grassland SOC stocks (12, 38). A recent study has reported that areas affected by multiyear droughts (MYDs) have increased by ~49,300 km^2^ annually since 1980, with grasslands experiencing the largest decline in vegetation (8). While the effects of short-term drought may be offset by the compensatory root growth, our results suggest that prolonged extreme drought weakens ecosystem resilience, causing profound declines in soil N availability, microbial functioning, and mineral protection. These changes could shift grasslands from soil C sinks to sources at long time scales. However, current Earth System Models (ESMs) often overlook the cumulative effects of MYDs (32). Our findings highlight the need for ESMs to incorporate the long-term impacts of extreme drought to better predict SOC vulnerability in future climate extremes.

A key uncertainty in soil C–climate feedback is how the subsoil responds to climate change (50). Our study highlights an overlooked risk of substantial subsoil C losses under extreme drought, which has not been reported previously. Although subsoil holds over half of the total SOC in grasslands, most field studies and models focus exclusively on surface layers. This is based on the assumption that soil C dynamics are mainly controlled by shallow roots and microbial activities (20, 51). Our study challenges this assumption by demonstrating significant subsoil C losses, particularly MAOC reductions, under multiyear extreme drought. These findings emphasize the critical role of subsoils in the long-term C stabilization. However, most ESMs oversimplify subsoil carbon by treating it as static and ignoring vertical heterogeneity in SOC responses to climate change (51). This oversimplification may lead to underestimates of long-term C losses under intensifying drought regimes. We therefore call for enhanced monitoring and a more mechanistic understanding of subsoil processes, as well as their incorporation into ESMs.

From a management perspective, protecting subsoil C is crucial for maintaining grassland C sinks in future drought scenarios. Our findings suggest that effective management strategies should address the mechanisms underlying subsoil C loss. First, improving soil nutrient availability could alleviate the microbial “N mining” of MAOC during drought. Second, management practices that enhance soil aggregation and mineral protection, such as applying organic amendments or reducing soil disturbance, could further stabilize MAOC. Third, sustaining plant diversity and promoting deep-rooting species can support the microbial precursors that are essential for MAOC formation. Integrating these strategies, which are based on the underlying mechanisms, into grassland management could reduce the long-term risk of subsoil C depletion under increasingly frequent and severe drought. While our results highlight the vulnerability of subsoil carbon to long-term extreme drought in alpine grasslands, it is unclear whether these findings can be generalized to ecosystems with different soil properties, vegetation compositions, or climatic regimes due to the scarcity of depth-resolved subsoil data. Moreover, the specific duration threshold at which extreme drought induces substantial subsoil carbon losses, along with the associated temporal dynamics, could not be fully resolved by our end-point sampling strategy (52). Addressing these uncertainties will require long-term drought-gradient experiments across diverse ecosystems with depth-resolved observations.

## Materials and Methods

### Study Site and Experimental Design.

We conducted an artificial rainfall gradient experiment to simulate drought effects in an alpine meadow located in Hongyuan County, Sichuan Province, China, on the eastern edge of the Tibetan Plateau (longitude 101°51′–103°23′ E, latitude 31°50′–33°22′ N, 3,500 m a.s.l.). This region is influenced by the Baihe River watershed, which experiences substantial seasonal groundwater fluctuations. Groundwater depth in this area varies from 43.5 ± 4.0 cm to 90.0 ± 0.5 cm (49). According to climate data from local meteorological stations (1961 to 2018), the annual mean temperature is 1.5 °C, and average annual precipitation is 747 mm, with approximately 80% of rainfall occurring from May to September (37). The experiment was established in 2014, and observations of most parameters started in 2016. We applied a completely randomized block design with six precipitation gradients: 5/4 P, P, 3/4 P, 1/2 P, 1/4 P, and 1/12 P, where P represents the ambient precipitation. Each plot measured 2 × 3 m and was separated by 2 m buffer zones to minimize interference between treatments. To prevent lateral water movement, iron plates were installed around the perimeter of each plot to a depth of 40 cm (Fig. 1*A*). Rainfall reduction was achieved by covering the plots with V-shaped or corrugated transparent plastic strips, with coverage adjusted according to the specific treatment. For instance, in the 3/4 P treatment, 25% of the plot area was covered. To avoid the potential effects of the plastic cover, inverted plastic strips were placed over the P and 5/4 P plots with a coverage of 50%. In addition, rainwater collected from the 3/4 P plots was promptly added to 5/4 P plots following each precipitation event during the growing season. This experimental design has been widely used for drought manipulation experiments in terrestrial ecosystems (53–55), and it effectively modulates rainfall across treatments (*SI Appendix*, Fig. S1). In this study, P, 1/2 P, 1/4 P, and 1/12 P treatments were selected to examine drought effects on SOC across soil profiles while minimizing cost and workload.

### Soil Sampling and Property Measurements.

After 10 y of drought treatments, soil samples were collected from five replicated control and drought plots during the peak growing season of 2023. Soil cores were extracted using an 8 cm diameter stainless steel corer to a depth of 60 cm in each plot, with five cores taken per plot. The soil below 60 cm was predominated by coarse sand with minimal organic matter content and was therefore excluded from sampling. Visible plant materials and stones were removed from each soil sample. For plant root density measurement, roots from one core per plot were isolated by soil depth. Soil samples were sectioned into six depth intervals: 0 to 10 cm, 10 to 20 cm, 20 to 30 cm, 30 to 40 cm, 40 to 50 cm, and 50 to 60 cm layers. End-point, depth-resolved sampling was used primarily due to the relatively small plot size and the destructive nature of deep coring, which would compromise plot integrity and the continuity of our long-term experiment. Considering these constraints and the slow turnover rate of SOC, particularly in deeper layers, this approach is suitable for capturing the cumulative effects of prolonged drought, as emphasized by a recent global synthesis (27). Soil samples were thoroughly mixed and sieved through a 2 mm mesh. Portions of each sample were stored at 4 °C for the analysis of microbial biomass carbon and nitrogen and microbial carbon use efficiency. Additional portions of soil samples were stored at −80 °C for microbial DNA extraction, while the remaining samples were air-dried for physicochemical property assessments, including carbon density, lignin phenol content, and amino sugar concentrations. To minimize disturbance to the experimental plots, undisturbed soil samples for bulk density determination were collected from each depth interval using a custom-made steel corer with an internal diameter matching that of a 100 mL ring knife. For subsequent SOC fractionation and mechanistic analyses, we focused on three representative soil layers (0 to 10 cm, 20 to 30 cm, and 40 to 50 cm), following the approach of Soong et al. (56). Our depth-resolved SOC results, which showed that the 20 to 30 cm and 30 to 40 cm subsoil layers exhibited similar and significant SOC declines under drought, whereas SOC stocks in the topsoil at 0 to 20 cm and deeper soil at 40 to 60 cm remained relatively stable. Accordingly, the 20 to 30 cm layer was selected to represent the subsoil, while the 0 to 10 cm and 40 to 50 cm layers represented the topsoil and deep soils, respectively. Given the labor-intensive nature of fractionation and the cost of microbial and mineral analyses, this representative-layer approach ensured analytical feasibility while capturing the most pronounced drought responses across soil profiles.

### Plant C Input.

Aboveground and belowground net primary productivity (ANPP and BNPP) were measured in each plot during the peak biomass (August) in each year. For ANPP, a 0.5 × 0.5 m quadrat frame was used to harvest aboveground biomass, separating live plant tissue and dead material by species. These samples were oven-dried at 65 °C for 48 h to a constant weight, and species richness was also recorded. BNPP was estimated using the ingrowth core method. Soil cores (8 cm in diameter, 40 cm deep) were taken from fixed positions within each plot. Live roots were separated and oven-dried at 70 °C for 48 h to a constant weight. After removing the soil cores, each hole was immediately refilled with sieved, root-free soils. The following year, soil cores were extracted from the same locations, following the same procedure, and each hole was refilled after sampling to allow for repeated annual measurements. The measurements of ANPP and BNPP started in 2016 and detailed methods are described in previous studies (57, 58). The plant C input to different soil layers was determined based on the measured ANPP, BNPP, and plant root density, calculated as follows.

$$\mathrm{Plant}\;C\;{\mathrm{input}}_{0-10}=\mathrm{ANPP}+\mathrm{BNPP}*f_{0-10},$$

$$\mathrm{Plant}\;C\;{\mathrm{input}}_{20-30}=\mathrm{BNPP}*f_{20-30},$$

$$\text{Plant}\;C\;{\text{input}}_{40-50}=\text{BNPP}*f_{40-50}.$$

where *f* represents the proportion of plant root distribution in specific soil layers.

### Soil Organic Carbon and Its Fractions.

SOC concentration was determined through titration after oxidation of soil samples with potassium dichromate and concentrated sulfuric acid. SOC stocks for each plot were calculated by multiplying the soil bulk density (g soil cm^−3^) by the SOC concentration (g C g soil^−1^) and the thickness of each soil layer (10 cm). A particle size fractionation method was used to separate particulate organic carbon (POC), which is less decomposed and unprotected by minerals, from mineral-associated organic carbon (MAOC), which is protected by mineral (59, 60). In brief, 10 g of soil was mixed with 30 mL of sodium hexametaphosphate (5 g L^−1^) and shaken for 24 h on an overhead shaker to disperse aggregates. The dispersed mixture was then thoroughly rinsed through a 53 µm sieve using an automated wet-sieving system, which separated POC (>53 µm) and MAOC (<53 µm) fractions. The separated fractions were dried at 60 °C, weighed, and then finely ground using a ball mill. The C content in the POC and MAOC fractions was determined through dichromate oxidation titration after drying and grinding. The citrate-bicarbonate-dithionite (CBD) extraction method was applied to isolate OC bound to reactive Fe and Al oxides (61). An additional aliquot was extracted with NaCl as a control. The amount of active iron-bound organic carbon (OC-Fe) was calculated as the difference in OC content between the NaCl- and dithionite-extracted soil residues and expressed as a percentage of total SOC by volume.

### Soil Physicochemical and Microbial analysis.

Aliquots of each soil sample were oven-dried at 105 °C to a constant weight to determine soil moisture. Soil pH was measured using a glass electrode (Mettler-Toledo, Zurich, Switzerland) in a 1:2.5 (w:v) soil-to-water suspension. Soil organic C and N were determined on air-dried, ground soil samples using an Elementar Vario Max CN elemental analyzer (Elementar, Germany). Prior to analysis, soil samples were acidified with 1 M HCl to remove potential inorganic carbonates. Dithionite-extractable iron and aluminum (Fe_d_ and Al_d_), including short-range ordered (SRO) and some crystalline forms, were extracted using a dithionite-citrate-bicarbonate (DCB) extraction method (62). Oxalate-extractable Fe and Al (Fe_o_ and Al_o_), which represents SRO and organically complexed forms, were extracted using acid ammonium oxalate (AAO). Lignin phenols were isolated from air-dried soil samples using alkaline CuO oxidation (63). Microbial biomass carbon (MBC) and nitrogen (MBN) were measured using the chloroform fumigation extraction method (64). Amino sugars were extracted and analyzed following the method of Zhang and Amelung (65), with quantification of glucosamine (GluN), galactosamine (GalN), and muramic acid (MurA). A substrate-independent method was employed to assess microbial carbon use efficiency (CUE) by tracking the incorporation of 18O from H2^18^O into microbial genomic DNA (66). Soil microbial DNA was extracted from fresh soil using the EZNA Soil DNA Isolation Kit (Omega Bio-Tek, Doraville, GA, USA). Specific methodology for the measurement of different indicators is provided in the supplementary information.

### Data Analysis.

The response of SOC stocks to drought treatments was calculated as the percentage difference between drought and control conditions, normalized by the control values: Drought response = (Drought − Control) / Control × 100. For other variables, we calculated the log response ratio (LnRR) to quantify drought effects: LnRR = ln (Drought/Control), which provides a standardized measure of proportional change. We used linear mixed-effects models in the lme4 and lmerTest packages to account for the hierarchical structure of the experimental design. Drought treatment was included as a fixed effect, while block was incorporated as a random effect. For variables (e.g., ANPP, BNPP, and plant species richness) that measured over multiple years, “year” and its interaction with drought treatment were also included as fixed effects to account for potential temporal variations. However, these interactions were not significant and thus were not presented in the *Results*. For significant treatment effects (*P* < 0.05), we performed post hoc pairwise comparisons using estimated marginal means with Tukey’s HSD adjustment for multiple comparisons (emmeans package in R). The interaction between treatment and soil depth or year was evaluated through the inclusion of an interaction term in the model. Model assumptions were verified through visual inspection of residual plots. Partial correlation analysis (ppcor package in R), Variance Partitioning Analysis (rdacca.hp package in R) and random forest analysis (rfPermute package in R) were employed to investigate the relative contributions of biotic and abiotic factors to variations in MAOC under drought conditions.

Amplicon sequence variant (ASV) data were filtered to exclude ASVs with relative abundances below 0.015% or those occurring in fewer than five samples per plot prior to constructing the microbial co-occurrence network. Spearman’s correlation coefficients and Jaccard dissimilarity P-values were calculated, combined using Brown’s method (67), and corrected for false discovery rate (FDR) following Benjamini et al. (68). Statistically significant associations (*P* < 0.05) were used to build the co-occurrence network. Network attributes, including network density, average degree, the number of positive and negative correlations, edges, and nodes, were extracted for each soil depth across plots (*SI Appendix*, Fig. S11 and Table S1). Principal component analysis (PCA; FactoMineR and factoextra packages) was used to reduce dimensionality in the attributes of microbial co-occurrence networks, with the first principal component (PC1) representing microbial structural and functional characteristics under different treatments.

## Supplementary Material

Appendix 01 (PDF)

## Data Availability

Experimental data used in this study are publicly available on Figshare (69). No custom code was used in this study. Other study data are included in the article and/or *SI Appendix*.
