# Supplementary material for: Decadal extreme drought reduces alpine subsoil carbon stocks
Source: Proc Natl Acad Sci U S A. 2026 Feb 20;123(8):e2517468123. doi: 10.1073/pnas.2517468123 (PMC12933107; doi:10.1073/pnas.2517468123)
Supplement: Supplementary file 1 — Appendix 01 (PDF) [file pnas.2517468123.sapp.pdf]

Supporting Information for

Decadal extreme drought reduces alpine subsoil carbon stocks

Ronglei Zhou[1,2,3], Jinsong Wang[1,2,3*], Quancheng Wang[1], Ning Liu[1], Chenglong Ye[4], Jingjing Shi[5], Mengjie Liu[1], Zhangwei Gao[6], Houkun Chu[1], Zhenrui Zhang[1], Bin Niu[1], Song Wang[1], Ruiyang Zhang[1,2], Dashuan Tian[1,2], Shuli Niu[1,2,3*]

[1]Key Laboratory of Ecosystem Network Observation and Modeling, Institute of Geographic Sciences and Natural Resources Research, Chinese Academy of Sciences, Beijing 100101, China

[2]Department of Environment and Resources, University of Chinese Academy of Sciences, Beijing 100049, China

[3]Sichuan Zoige Alpine Wetland Ecosystem National Observation and Research Station, Institute of Geographic Sciences and Natural Resources Research, Chinese Academy of Sciences, Beijing 100101, China

4College of Resources and Environmental Sciences, Nanjing Agricultural University, Nanjing 210095, China

[5]College of Forestry, Beijing Forestry University, Beijing 100083, China

[6]College of Juncao Science and Ecology, Fujian Agriculture and Forestry University, Fuzhou 350000, China

[*] Jinsong Wang, Shuli Niu

Email: wangjinsong@igsnrr.ac.cn, sniu@igsnrr.ac.cn

This PDF file includes:

Supporting text
Figures S1 to S11
Table S1
SI References

Supporting Information Text

Microbial biomass carbon (MBC) and nitrogen (MBN)

Microbial biomass carbon (MBC) and nitrogen (MBN) were measured using the chloroform fumigation extraction method (1). Two 10 g fresh soil samples were placed in 25 mL beakers. One sample was transferred to a desiccator containing a beaker with ethanol-free chloroform and a few boiling stones, as well as a small beaker with 1 mol L^{-1} NaOH solution. The desiccator was evacuated to induce chloroform boiling for 5 minutes and then sealed to incubate in the dark at 25°C for 24 hours. The second sample was placed in a separate desiccator as an unfumigated control. After fumigation, the vacuum was released, and the NaOH beaker was removed. The desiccator was then repeatedly evacuated to remove any residual chloroform odor from the soil. Both fumigated and non-fumigated soil samples were transferred to 50 mL polyethylene centrifuge tubes, and 40 mL of 0.5 mol L^{-1} K_2SO_4 was added to each tube. Samples were shaken at 800 rpm for 30 minutes and subsequently filtered. A 10 mL aliquot of the extract was analyzed for MBC and MBN using a total organic carbon analyzer (vario TOC cube, Elementar, Germany).

Lignin phenols

Lignin phenols were isolated from air-dried soil samples using alkaline CuO oxidation (2). Briefly, for each sample containing less than 5 mg of organic carbon, 10 mg of glucose was added to stabilize the carbon content and minimize lignin phenol losses during oxidation. A mixture containing 1 g soil, 1 g CuO, 0.1 g ammonium iron (II) sulfate hexahydrate, and 15 mL of 2 M NaOH was heated to 170 °C for 2.5 h and then cooled to room temperature. Ethylvanillin (40 μg) was added as an internal standard, and the mixture was centrifuged at 4,000 rpm for 3 minutes. The pH of the supernatant was adjusted to 1.8–2.0 using 6 M HCl, and lignin oxidation products were isolated by centrifugation at 4,000 rpm for 10 minutes. The supernatant was transferred to a clean tube, mixed with 10 mL of ethyl acetate, and the ethyl acetate layer was dried under a stream of N2 at 38°C. The residue was then dissolved in pyridine and N,O-bis-(trimethylsilyl) trifluoroacetamide and derivatized at 70°C for 3 hours. Lignin phenol derivatives were quantified using a TRACE 1300 gas chromatograph equipped with a TG-Sil-5MS column (Thermo Fisher Scientific, Waltham, MA, USA). Lignin phenols included cinnamyl (e.g., ferulic and p-coumaric acids), guaiacyl (e.g., vanillin, acetovanillone, and vanillic acid), and syringyl (e.g., syringaldehyde, acetosyringone, and syringic acid) compounds. Total lignin phenol content was defined as the sum of cinnamyl, guaiacyl, and syringyl components.

Amino sugars

Amino sugars were extracted and analyzed following the method of Zhang and Amelung (3), with quantification of glucosamine (GluN), galactosamine (GalN), and muramic acid (MurA). MurA serves as a specific indicator of bacterial necromass, while fungal-derived GluN (FGluN) was calculated by subtracting bacterial-derived GluN from the total GluN, as GluN is present in both fungal and bacterial cell walls. Fungal GluN was determined as follows: FGluN (μg/g) = (GluN/179.17 - 2 × MurA/251.23) × 179.17, where 179.17 and 251.23 are the molecular weights of GluN and MurA, respectively. Science the origin of GalN could not be reliably distinguished between microbial sources, it was not analyzed further in this study. Fungal and bacterial necromass carbon (C) were calculated based on the concentration of FGluN and MurA, respectively, with the following conversion factors: fungal necromass C (μg/g soil) = FGluN × 9 and bacterial necromass C (μg/g soil) = MurA × 45. Here, 9 and 45 are the conversion factors that translate GluN and MurA into fungal and bacterial necromass C, respectively.

Microbial carbon use efficiency (CUE)

We employed a substrate-independent method to assess microbial growth by tracking the incorporation of ^{18}O from $H_2{}^{18}O$ into microbial genomic DNA (4). 10 g of fresh soils were placed in 50 mL tubes and pre-incubated in the dark at 25°C for 7 days, maintaining soil moisture at 60% of

water-holding capacity (WHC). Following pre-incubation, each soil sample (600 mg) was divided into two portions and placed in 2 mL amber chromatography vials. One proportion served as a control for natural ^{18}O abundance, while the other was designated as the labeled sample. For one of the replicate samples, ^{18}O enrichment was achieved by adding water with ^{18}O to adjust soil water to 20.0 at% ^{18}O, while the other received an equivalent volume of unlabeled water. Additional Millipore ultrapure water was added to maintain 60% WHC. Vials were then placed in 20 mL headspace containers, with three empty control vials included for each batch of samples.

After 24 hours of incubation at 25°C in the dark, microbial respiration was monitored during incubation, and 12 mL of headspace gas was sampled from each vial at the end of the incubation. The removed volume was replaced with 10 mL of air with a known CO_2 concentration. CO_2 concentrations were measured using gas chromatography (GC-7890B, Agilent, California, USA). Microbial respiration rate was calculated as hourly CO_2 production per gram of soil dry weight over the 24-hour incubation period:

$$Respiration = \frac{Rs}{DW \times t} \times \frac{p \times n}{R \times T} \times V \times 1000$$

where p is the atmospheric pressure (kPa), n is the molecular mass of C (12.01 g mol^{-1}), R is the ideal gas constant (8.314 J mol^{-1} K^{-1}), and T is the absolute temperature of the gas (295.15 K). V is the volume of headspace of the vial (L). Rs (ppm) is the concentration of CO2 produced in the 24 h incubation period.

After gas collection, the brown chromatography vials were transferred to liquid nitrogen for freezing and stored at −20 °C for subsequent DNA extraction. DNA was extracted from both labeled and unlabeled soil samples extracted using a DNA extraction kit (MoBio, Powersoil). DNA concentration was quantified using the PicoGreen® assay (Quant-iTTM PicoGreen® dsDNA reagent, Life Technologies). For isotope analysis, the remaining DNA extracts were transferred to silver capsules, dried at 45°C for 5 hours to remove moisture, and analyzed for ^{18}O and ^{16}O abundances using isotope ratio mass spectrometry (IRMS-TC/EA; Thermo Scientific) to measure 18O enrichment and total oxygen content.

Microbial community growth (Growth; µg C g^{-1} soil h^{-1}) and microbial carbon use efficiency (CUE) were determined by calculating the DNA production rate (DNAp; µg DNA g^{-1} soil h^{-1}) over the incubation. DNAp was estimated by multiplying the total oxygen content (O_{Total}) by the ^{18}O excess of DNA relative to the unlabeled DNA samples ($at\%_{excess}$):

$$DNA_P = O_{total} \times (\frac{at\%_{excess}}{at\%_{begin}} \times 100)/31.21$$

where $at\%_{begin}$ is the ^{18}O at% of soil water at the beginning of incubation (20.0% in this study). A conversion factor (fDNA) was calculated as the ratio of MBC to DNAp. Growth was calculated based on the DNAp and fDNA:

$$Growth = \frac{f_{DNA} \times DNA_p}{DW \times T}$$

where DW is the dry weight of soil sample and T is the incubation time. To obtain microbial community CUE, we divided microbial growth by total carbon uptake (U; expressed as µg carbon per hour per gram of soil dry mass), which was calculated as the sum of microbial growth and respiration:

$$CUE = \frac{Growth}{Growth + Respiration}$$

Soil microbial DNA

Soil microbial DNA was extracted from 0.25 g of fresh soil using the EZNA Soil DNA Isolation Kit (Omega Bio-Tek, Doraville, GA, USA), followed by further purification with the DNeasy PowerClean Pro Cleanup Kit (Qiagen, Hilden, Germany). The extracted DNA was separated and identified on a 1% agarose gel, and DNA concentration and purity were assessed using a NanoDrop UV-Vis spectrophotometer (ND-2000c; NanoDrop Technologies, Wilmington, DE, USA). PCR amplification of the bacterial 16S rRNA gene (V3-V4 region) and fungal ITS gene (ITS1 region) was performed using the primer pairs 338F/806R and ITS1F/ITS2-2043R, respectively. High-throughput

sequencing of PCR amplicons was conducted on an Illumina MiSeq platform (Novogene, Beijing, China).
Sequence data were processed and quality controlled using QIIME 1.9.1, following the protocol of Caporaso et al. (2010) (5). Briefly, sequences with an average quality score below 20 over a 50 bp sliding window, shorter than 50 bp, or containing ambiguous bases were removed. Duplicates longer than 10 bp were merged, unmergable sequences were discarded, and samples were distinguished based on barcode and primer sequences with direction corrected. Amplicon sequence variants (ASVs) were identified at 100% similarity using UPARSE, with chimeras filtered out. Taxonomic assignments for bacterial 16S rRNA and fungal ITS2 sequences were conducted using the SILVA (SILVA 132) and UNITE (version 7.2) database, respectively. The resulting ASV tables with taxonomic annotations were then for used downstream analyses.

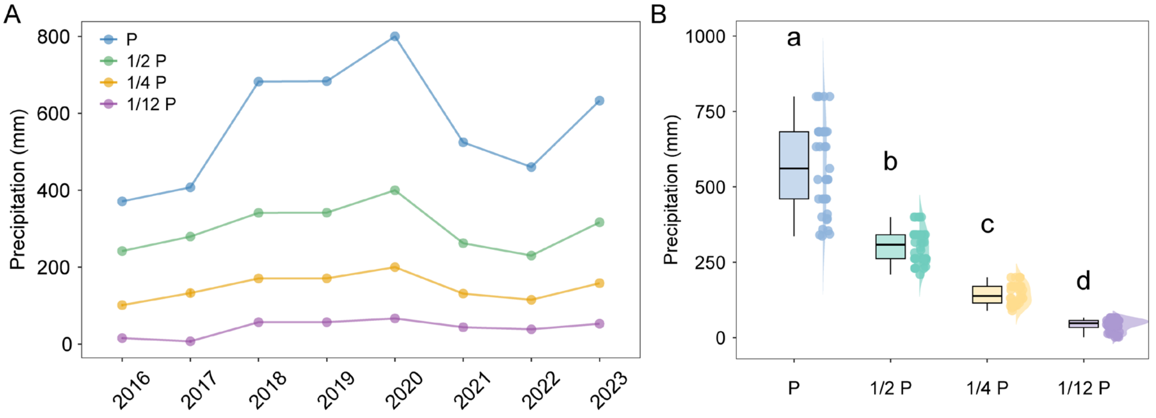

Fig. S1. Multi-year average rainfall dynamics across drought gradients. A, Inter-annual variability of rainfall under different drought treatments. **B**, Differences in rainfall among drought treatments over the course of the experiment. P in the X axis represents the ambient precipitation. Box boundaries represent the 75th and 25th quantiles, and whiskers represent 1.5 times the interquartile range. The scatter plot represents the observations and the black line in the boxplot is the median of the data for each treatment. Different lowercase letters on the top of bars indicate significant differences among drought treatments. Statistical analysis was performed by linear mixed-effects models at the significance level of 0.05.

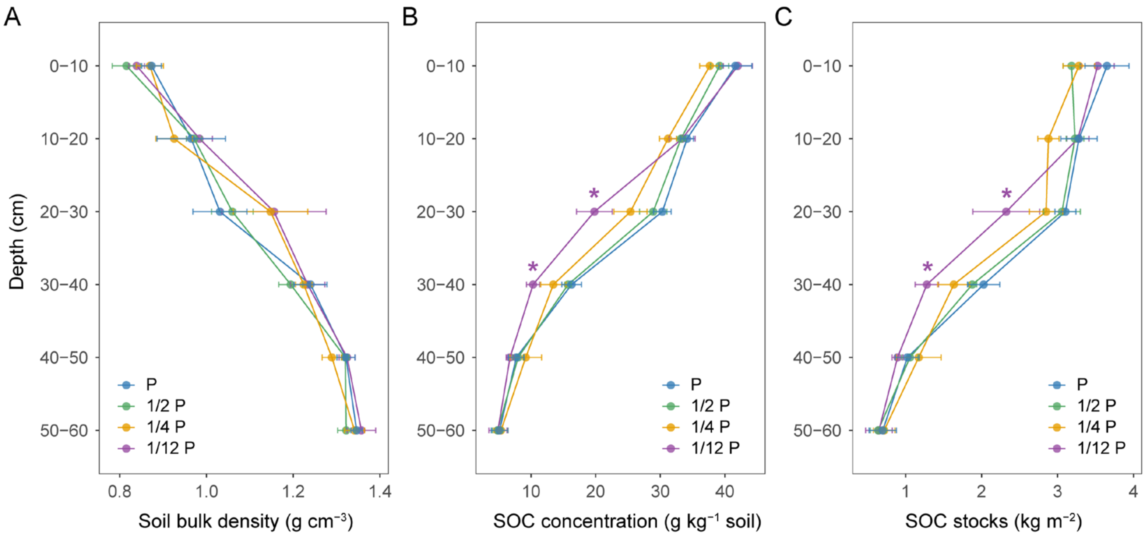

Fig. S2. Changes in soil bulk density (A), SOC concentration (B), and SOC stocks (C) across soil profiles under different drought treatments. Data are mean values ± s.e.m (*n* = 5). *P* is ambient precipitation. *represents significant difference between drought treatment and ambient at *P*<0.05.

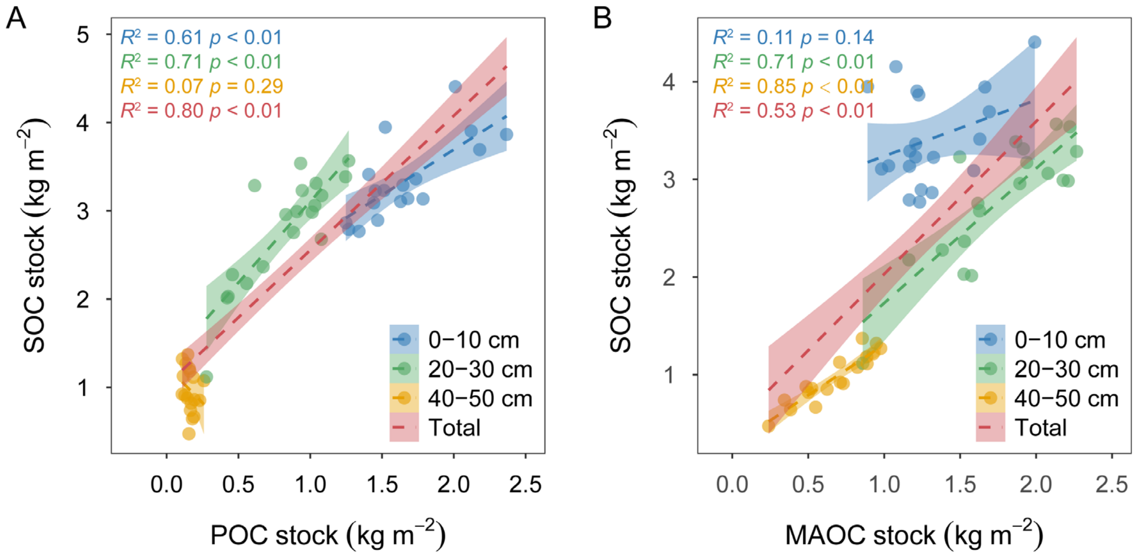

Fig. S3. Relationships between the stocks of SOC and its fractions at different soil depths. **A**, The relationship between soil particulate organic C (POC) and SOC stock in the topsoil (0–10 cm), subsoil (20–30 cm), deep soil (40–50 cm) and across different soil depths. **B**, The relationship between soil mineral-associated organic C (MAOC) and SOC stock in different soil depths. The dashed line represents the fitted line and the shaded area indicates 95% confidence intervals.

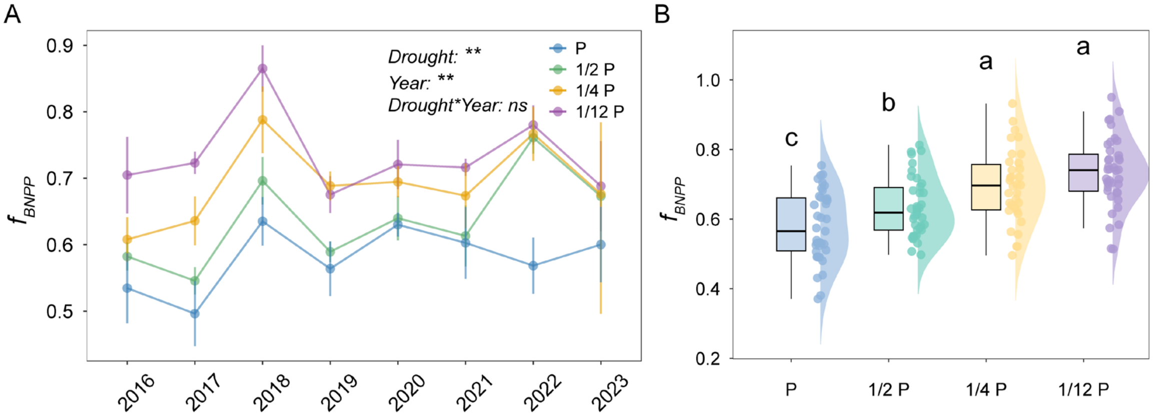

Fig. S4. Effect of drought on the ratio of belowground net primary productivity (BNPP) to plant net primary productivity (f_{BNPP} = BNPP/NPP). **A**, Temporal changes of f_{BNPP} under different drought treatments. **B**, Drought effects on mean f_{BNPP} over the experimental period. Box boundaries represent the 75th and 25th quantiles, and whiskers represent 1.5 times the interquartile range. Lines in the boxplot represent the median value. Data are mean values ± s.e.m (n = 5). Different lowercases letters on the top of bars represent significant differences among drought treatments. Statistical analysis was performed by linear mixed-effects models at the significance level of 0.05. $^{**}P$<0.01, $^{*}P$<0.05, ns: no significant effect.

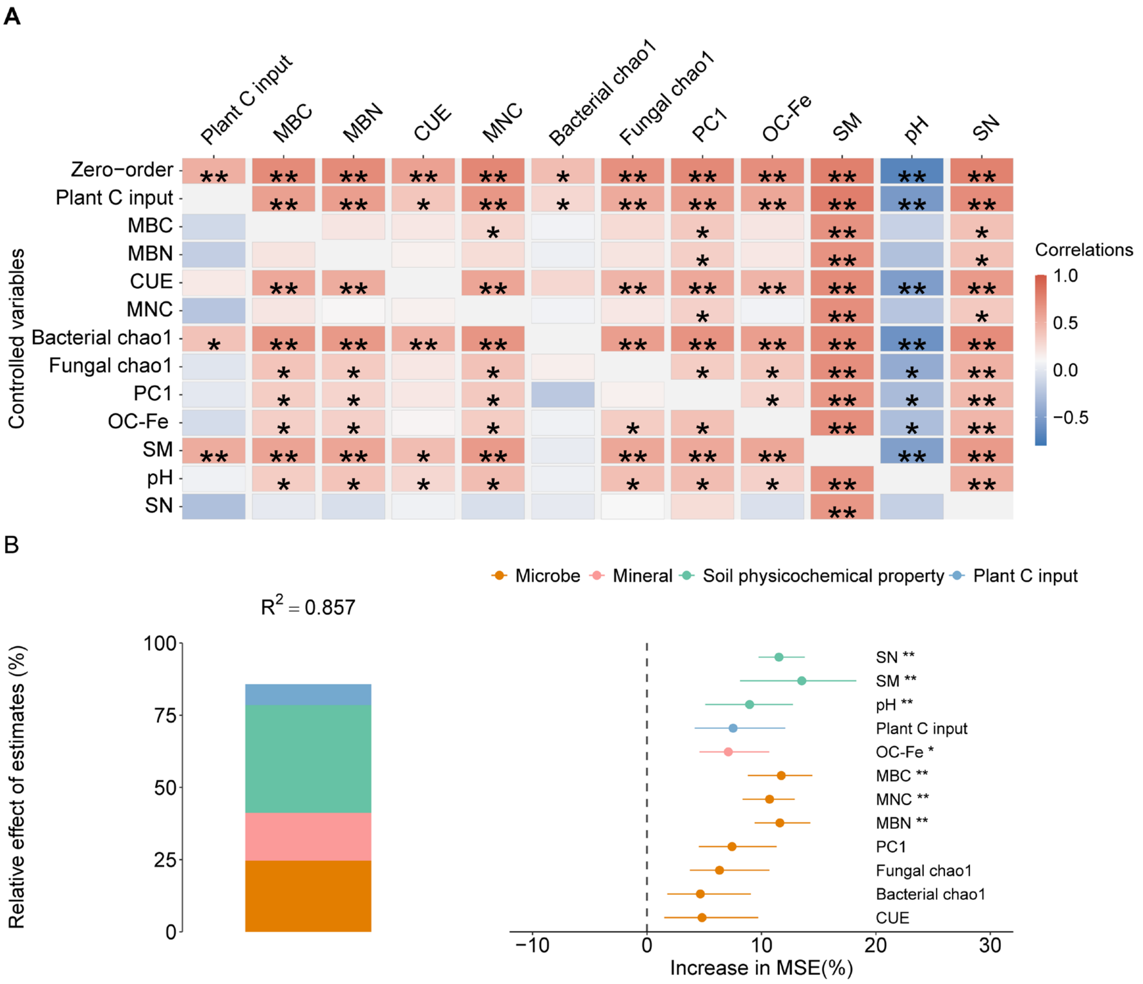

Fig. S5. Contribution of soil physicochemical properties, plant carbon input, microbial properties, and mineral factors to MAOC variations under drought conditions. **A**, Partial correlations between MAOC and controlling factors. The first row shows the factors for which correlations with MAOC are examined. The color of the square indicates the strength and sign of the correlation. **B**, Relative importance of different variables to the variation of MAOC in variation partitioning and random forest analysis. MBC: microbial biomass carbon; MBN: microbial biomass nitrogen; CUE: microbial carbon use efficiency; MNC: microbial necromass carbon; PC1: The first principal component from principal component analysis (PCA) downscaling of microbial co-occurrence network parameters; OC-Fe: Iron-bound organic carbon; SM: soil moisture, SN: total soil nitrogen. $^{**}P$ <0.01, $^{*}P$ < 0.05.

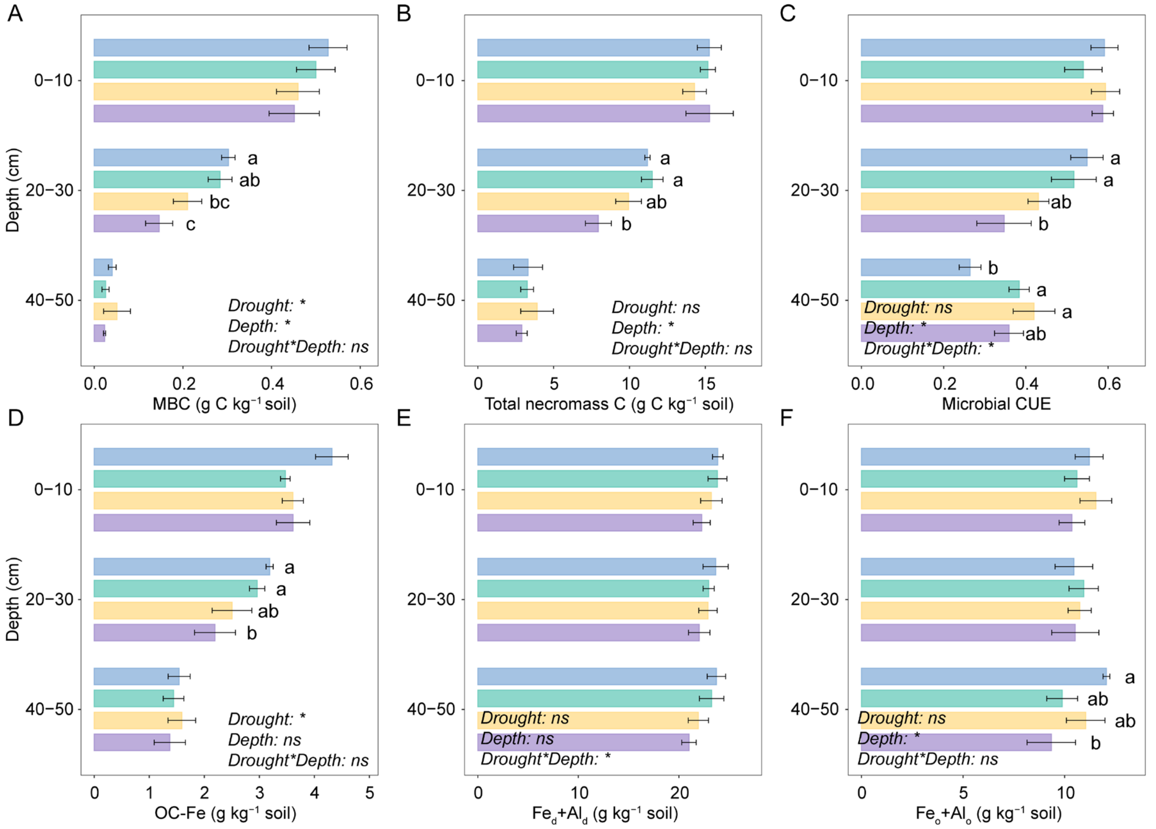

Fig. S6. Responses of soil microbial and mineral properties to drought in different soil depths. **A**, Effect of drought on soil microbial biomass carbon (MBC). **B**, Drought responses of microbial necromass carbon (MNC). **C**. Effect of drought on microbial carbon use efficiency (CUE). **D**, Response of iron-bound organic carbon (OC-Fe) to drought. **E**, Effect of drought on sum of dithionite-extractable Fe and Al content (Fe_d+Al_d). **F**, Effect of drought on sum of Oxalate-extractable Fe and Al content (Fe_o+Al_o). All these data were measured in 2023. Data are mean values ± s.e.m (n = 5). Different lowercase letters on the top of bars represent significant differences among drought treatments. Statistical analysis was performed by linear mixed-effects models at the significance level of 0.05. $^*P<0.05$, ns: no significant effect.

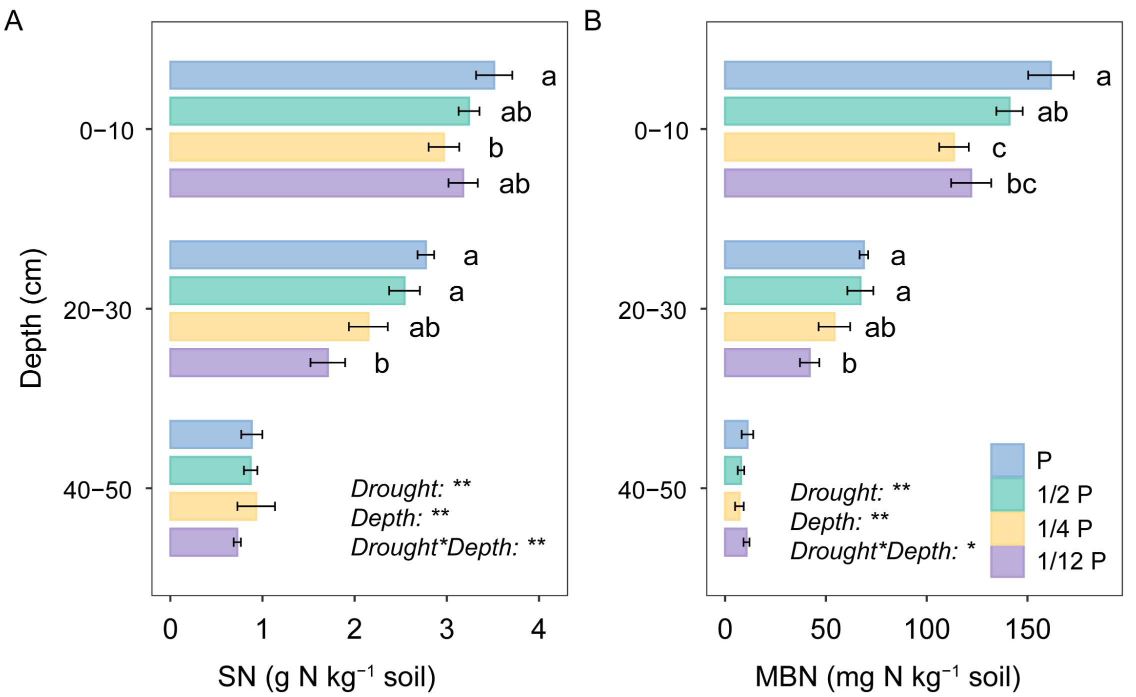

Fig. S7. Changes in soil total nitrogen and microbial biomass nitrogen in different soil depths under drought treatments. **A**, Drought effects on soil total nitrogen (SN). **B**, Drought effects on soil microbial biomass nitrogen (MBN). All these data were measured in 2023. Data are mean values ± s.e.m (n = 5). Different lowercase letters on the top of bars represent significant differences among drought treatments. Statistical analysis was performed by linear mixed-effects models at the significance level of 0.05. $^{**}P<0.01$, $^{*}P<0.05$.

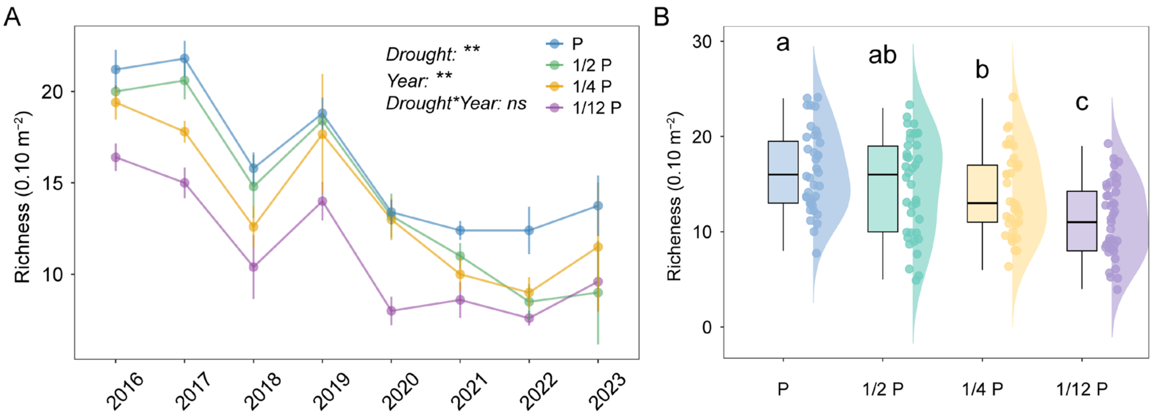

Fig. S8. Drought effects on plant diversity. **A**, Temporal changes of plant richness under different drought treatments. **B**, Drought effects on mean plant diversity over the experimental year. Box boundaries represent the 75th and 25th quantiles, and whiskers represent 1.5 times the interquartile range. Lines in the boxplot represent the median value. Data are mean values ± s.e.m (n = 5). Different lowercase letters on the top of bars represent significant differences among drought treatments. Statistical analysis was performed by linear mixed-effects models at the significance level of 0.05. $^{**}P$<0.01, $^{*}P$<0.05, ns: no significant effect.

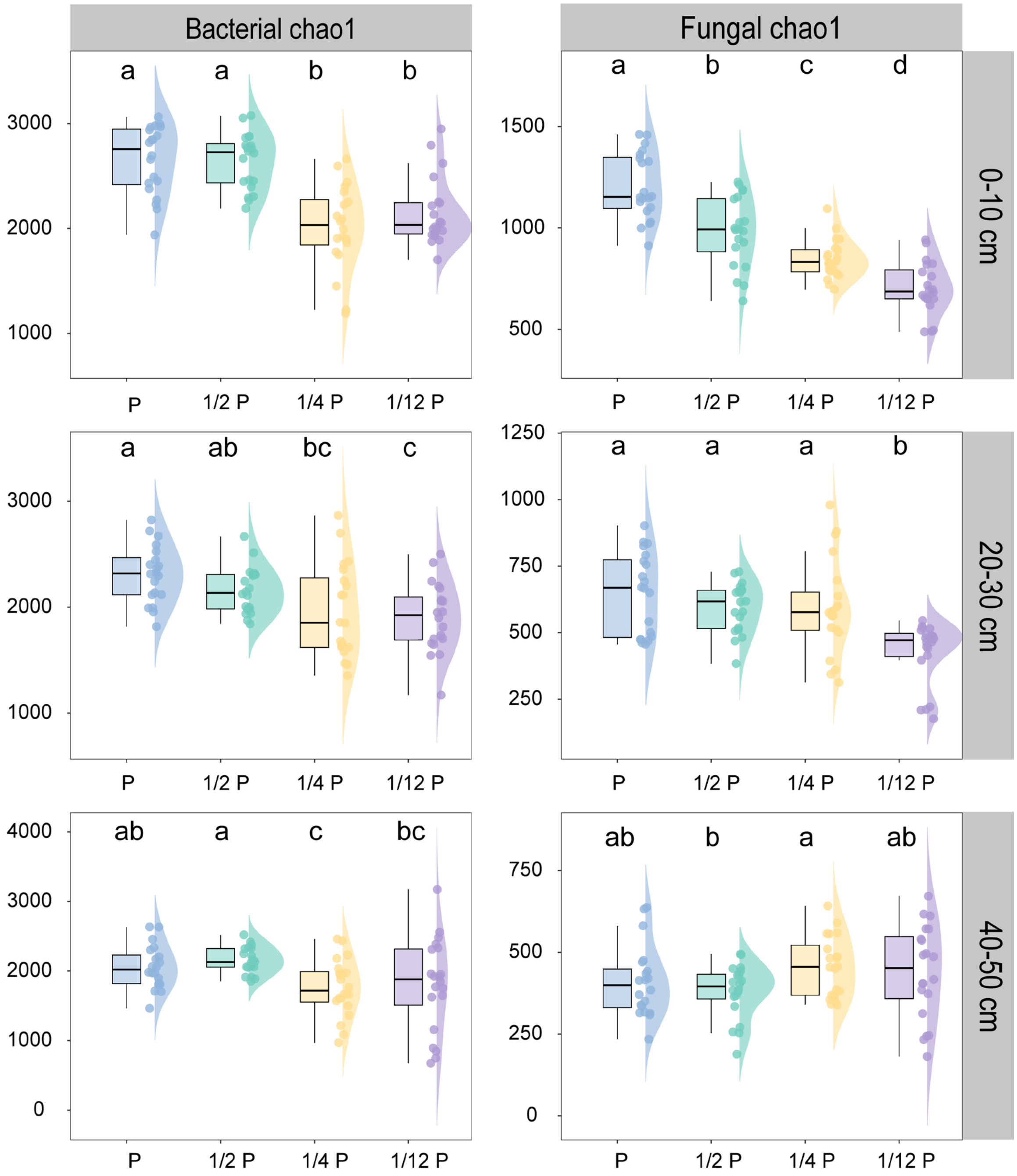

Fig. S9. Changes in soil bacterial and fungal diversity in different soil depths under drought treatments. Box boundaries represent the 75th and 25th quantiles, and whiskers represent 1.5 times the interquartile range. Lines in the boxplot represent the median value. Data are mean values ± s.e.m (n = 5). Different letters on the top of bars represent significant differences among drought treatments. Statistical analysis was performed by linear mixed-effects models at the significance level of 0.05.

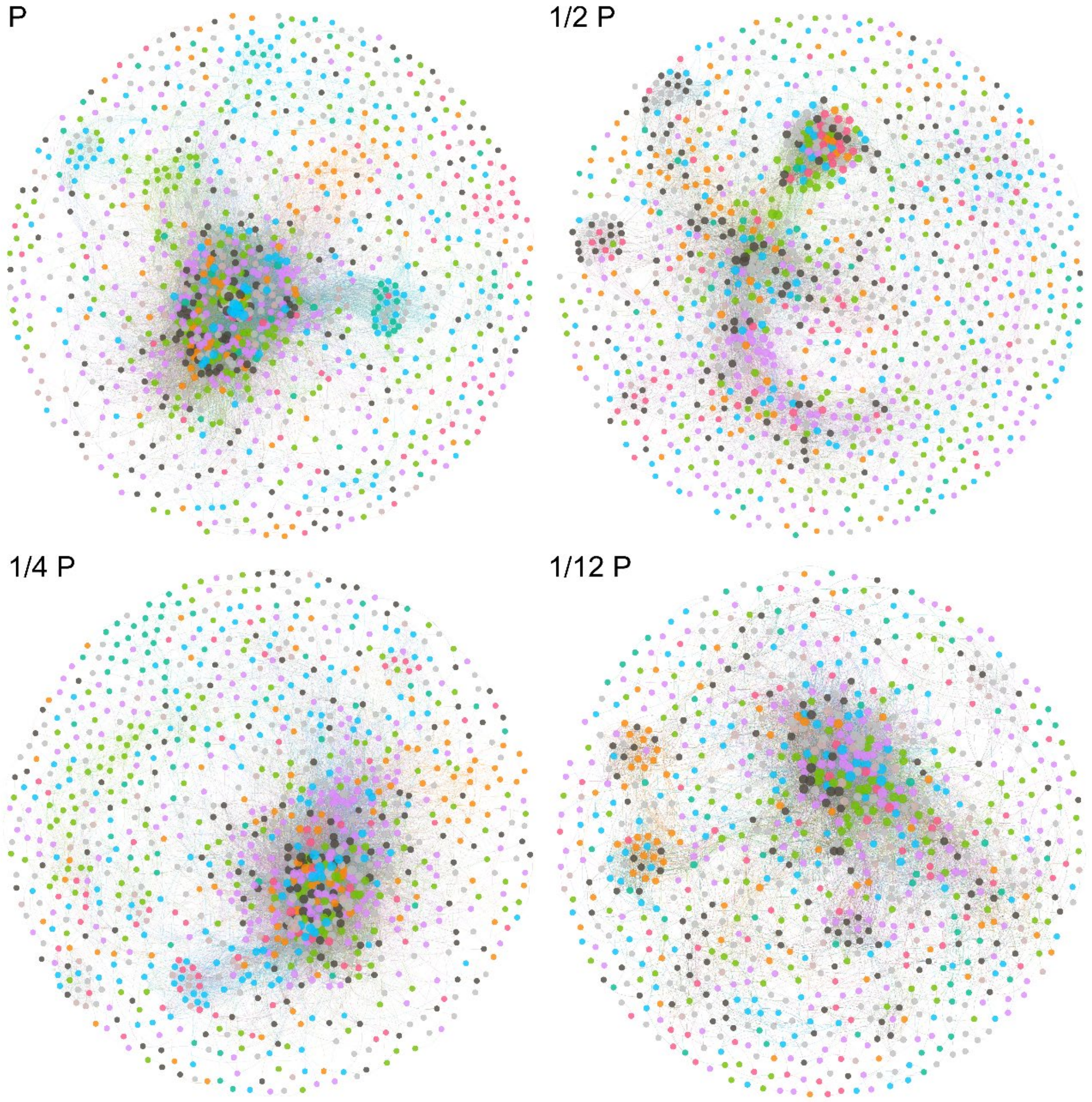

Fig. S10. Subsoil microbial co-occurrence network under different drought treatments. Each node represents a species or taxa and the size of each node is proportional to degree of the amplicon sequence variant (ASVs). Links indicate significant correlations between them (*P*<0.05). The colors of the nodes represent microorganisms at phylum levels.

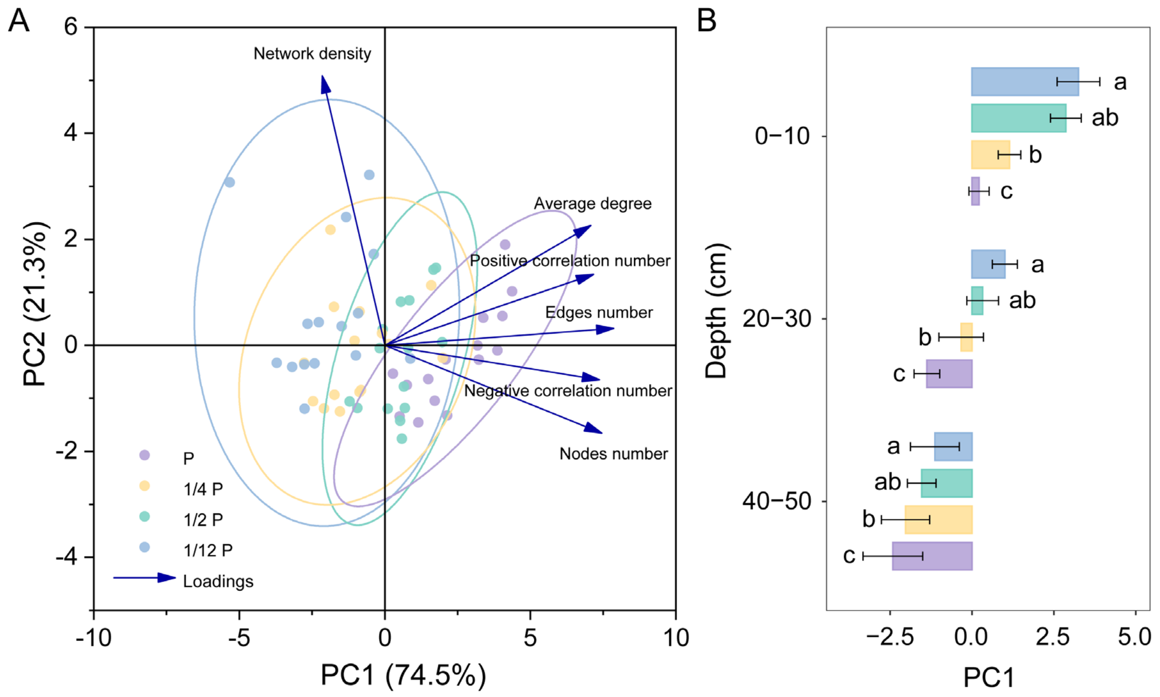

Fig. S11. Attributes of microbial co-occurrence network under different drought treatments. A, PCA downscaling results for different microbial co-occurrence network properties, including network density, average degree, positive and negative correlation numbers, edges, and nodes. **B**, Variations of principal component 1 (PC1) in different soil depths. Data are mean values ± s.e.m (*n* = 5). Different lowercase letters on the top of bars represent significant differences among drought treatments. Statistical analysis was performed by linear mixed-effects models at the significance level of 0.05.

Table S1. The properties of subsoil microbial co-occurrence network under different drought treatments.

Treatment	Nodes number	Edges number	Positive correlation number	Negative correlation number	Average degree	Average path length	Network diameter	Network density	Clustering coefficient
P	1147	28493	16794	11699	49.683	2.999	10.680	0.043	0.533
1/2 P	1171	13150	10130	3020	22.459	3.361	9.240	0.019	0.569
1/4 P	1094	21638	14027	7611	39.558	3.008	9.058	0.036	0.519
1/12 P	948	12258	8360	3898	25.861	3.096	9.066	0.027	0.491

The co-occurrence network properties include network density, average degree, positive and negative correlation numbers, edges, and nodes. P is the ambient precipitation.

SI References

1. P. C. Brookes, A. Landman, G. Pruden, D. S. Jenkinson, Chloroform Fumigation and the Release of Soil-Nitrogen - a Rapid Direct Extraction Method to Measure Microbial Biomass Nitrogen in Soil. Soil Biol Biochem 17, 837-842 (1985).
2. K. Kaiser, R. Benner, Characterization of Lignin by Gas Chromatography and Mass Spectrometry Using a Simplified CuO Oxidation Method. Anal Chem 84, 459-464 (2012).
3. X. D. Zhang, W. Amelung, Gas chromatographic determination of muramic acid, glucosamine, mannosamine, and galactosamine in soils. Soil Biol Biochem 28, 1201-1206 (1996).
4. E. Simon et al., Microbial growth and carbon use efficiency show seasonal responses in a multifactorial climate change experiment. Commun Biol 3, 584 (2020).
5. J. G. Caporaso et al., QIIME allows analysis of high-throughput community sequencing data. Nat Methods 7, 335-336 (2010).
